# Nanocomposite scaffolds in spinal fusion surgery: toward enhanced osteointegration and reduced inflammation

**DOI:** 10.1097/MS9.0000000000004519

**Published:** 2025-12-09

**Authors:** Hasibullah Aminpoor, Muhammad Zaib, Muhammad Khizar, Qaima Ali, Hasiba Karimi

**Affiliations:** a Faculty of Medicine, Kabul University of Medical Sciences “Abu Ali Ibn Sina”, Kabul, Afghanistan; b Faculty of Medicine, Georgian American University, Manifest Medical Research Co, Tbilisi, Georgia; c Faculty of Medicine, Liaquat College of Medicine & Dentistry, Karachi, Pakistan; d Faculty of Medicine, Bezmialem Vakif University, Istanbul, Turkey

**Keywords:** bone regeneration, inflammation, nanocomposite scaffolds, osteointegration, spinal fusion, tissue engineering

## Abstract

Spinal fusion surgery often fails when bone graft substitutes cannot achieve robust osteointegration or provoke inflammation. Nanocomposite scaffolds such as hybrid biomaterials combining polymers, bioceramics, and nanofillers are emerging solutions. By mimicking bone’s extracellular matrix and releasing bioactive ions or growth factors, these scaffolds significantly boost new bone formation. Recent preclinical studies in diverse settings report much higher fusion rates using nanocomposite scaffolds. For example, magnesium-doped hydroxyapatite/collagen scaffolds supplemented with platelet-rich plasma achieved solid fusion in 75% of treated animals versus 12.5% controls. Similarly, rat models in Japan showed 91.6% fusion using nano-hydroxyapatite/polymer scaffolds with low-dose BMP-2. These innovations not only promote osteoblast differentiation and angiogenesis but also allow incorporation of anti-inflammatory components (e.g., cerium oxide or silver nanoparticles) to mitigate post-surgical immune reactions. As international research groups in China, Japan, Iran, and elsewhere advance these materials, ongoing emphasis on standardized reporting and transparency is crucial. Our analysis underscores that nanocomposite scaffolds can bridge nanotechnology with clinical spinal reconstructive practice, justifying further clinical translation.

*To the Editor,*

Spinal fusion is a cornerstone treatment for degenerative and deformity indications, yet nonunion (pseudoarthrosis) remains a common complication due to inadequate bone regeneration^[1]^. Traditional bone grafting and bone morphogenetic protein (BMP) therapies can be limited by donor morbidity and inflammation. Nanocomposite scaffolds, combining synthetic or natural polymers with nano-hydroxyapatite, bioactive glass, or metallic nanoparticles, are designed to overcome these issues. Their nanoscale features and chemistries closely mimic bone matrix, enhancing osteoblast attachment and differentiation.

For instance, scaffolds doped with ultrafine cerium oxide nanoparticles have been shown to upregulate osteogenic pathways (via extracellular matrix and PI3K–Akt modulation) while reducing oxidative stress in intervertebral fusion models^[1]^. Similarly, titanium implants coated with nanotextured layers such as TiO_2_ or bioglass dramatically improved osteoblast activity and early fusion rates in animal studies^[2,3]^. These surfaces also enable incorporation of antimicrobial agents; composites with silver nanoparticle–peptide coatings demonstrated strong bactericidal action and reduced inflammatory markers *in vivo*^[2,4]^.

Recent innovations illustrate the clinical potential of nanocomposite grafts. In the United States, Van Eps *et al* reported that a magnesium-doped hydroxyapatite/collagen scaffold augmented with autologous platelet-rich plasma nearly doubled new bone volume and achieved a 75% fusion rate, far surpassing controls^[5]^. In Japan, a biodegradable composite of nano-hydroxyapatite and poly(lactic acid)–poly(ethylene glycol) (PLA–PEG) polymer enabled robust L4–L5 fusion in 91.6% of rats using a low dose of BMP-2 (compared to 0% in untreated controls)^[6]^. Chinese researchers have similarly enhanced scaffold performance by embedding cerium oxide nanoparticles into allograft matrices, yielding excellent bone ingrowth and anti-inflammatory benefits^[1]^.

European and North American studies now highlight the importance of controlled porosity and ion release: zinc- and copper-doped nanocomposites promote angiogenesis and osteogenesis by activating the HIF-1α/VEGF axis^[7]^, while graphene oxide–reinforced 3D-printed scaffolds improve compressive strength and osteointegration^[8]^. Reviews from Iran and elsewhere note that adding magnetic nanoparticles or carbon nanofibers to composite scaffolds stimulates mesenchymal cell osteogenesis and angiogenesis in bone defects^[9,10]^. Together, these examples underscore a global movement toward biomimetic, nanotechnology-driven solutions in spine surgery.

In summary, nanocomposite scaffolds represent a promising convergence of tissue engineering and spinal surgery. By enhancing osteointegration through biomimetic mineralization and by attenuating inflammation via antioxidant or antimicrobial additives, these materials address key pathophysiologic challenges in fusion. Ongoing studies must refine scaffold design (porosity, bioactive agent release, and mechanical strength) and validate safety *in vivo*. Clinicians should watch for emerging clinical trials of 3D-printed nanocomposite cages and hybrid grafts. Importantly, as computational modeling and AI increasingly support scaffold design and analysis, rigorous adherence to transparent reporting is needed. These guidelines reinforce that the development of novel spinal implants should be accompanied by clear disclosure of methods, ensuring reproducibility and trust in new technologies.

Our work is in line with the TITAN Guidelines on the need for transparency in AI use in healthcare^[11]^.

## Data Availability

Not applicable.
